# Afterload dependence of right ventricular myocardial deformation: A comparison between tetralogy of Fallot and atrially corrected transposition of the great arteries in adult patients

**DOI:** 10.1371/journal.pone.0204435

**Published:** 2018-09-27

**Authors:** Aleksandra Trzebiatowska-Krzynska, Eva Swahn, Lars Wallby, Niels Erik Nielsen, Carl Johan Carlhäll, Lars Brudin, Jan E. Engvall

**Affiliations:** 1 Department of Cardiology and Department of Medical and Health Sciences, Linkoping University, Linkoping, Sweden; 2 Department of Clinical Physiology and Department of Medical and Health Sciences, Linkoping University, Linkoping, Sweden; 3 Department of Clinical Physiology, Kalmar County Hospital and Department of Medical and Health Sciences, Linkoping University, Linkoping, Sweden; 4 Centre for Medical Image Science and Visualization (CMIV), Linkoping University, Linkoping Sweden; Faculty of Medical Science - State University of Campinas, BRAZIL

## Abstract

**Background:**

Prior studies suggested that myocardial deformation is superior to conventional measures for assessing ventricular function. This study aimed to evaluate right ventricular (RV) myocardial deformation in response to increased afterload. Patients with the RV in the systemic position were compared with patients with the RV in the sub-pulmonic position with normal or only slightly elevated systolic right ventricular pressure. Correlations between global longitudinal strain (GLS), radial strain, atrioventricular plane displacement (AVPD), and exercise capacity were evaluated.

**Methods:**

44 patients with congenital heart defect were enrolled in the study. The control group consisted of seven healthy volunteers. All patients underwent cardiovascular magnetic resonance (CMR) and cardiopulmonary exercise testing. We assessed biventricular myocardial function using CMR based feature tracking and compared the results to anatomic volumes.

**Results:**

Strain analysis and displacement measurements were feasible in all participants. RVGLS and RVAVPD were reduced in both study groups compared to the control group (p<0.001). Left ventricular (LV) radial strain was significantly lower in patients with a systemic RV than in those with a subpulmonic RV and lower than in controls (p<0.001). Both LVAVPD and RVAVPD were significantly depressed in patients compared to controls (p<0.05). RVAVPD was more depressed in patients with a high systolic RV pressure than in those with normal RV pressure (p<0.001). RVAVPD did not correlate with exercise capacity in either study group. Exercise capacity in both patient groups was depressed to levels reported in previous studies, and did not correlate with RVGLS.

**Conclusions:**

Both study groups had abnormal myocardial deformation and increased RV volumes. RVGLS in patients was lower than in controls, confirming the effect of increased afterload on myocardial performance.

## Introduction

Right ventricular (RV) function exerts an important influence on morbidity and mortality in patients with heart disease [1] [2]. Research over the last few decades has revealed the importance of RV function for prognosis [3] [4], particularly in persons with CHD (Congenital Heart Disease) as those patients are often young and face repeated cardiac surgery. Assessment of RV function is challenging due to the crescentic shape of the RV and its central position in the thoracic cavity complicating visualization with ultrasound [5], especially in case of congenital cardiac malformations and after surgery [6] [7] [8]. Measurements of RV volume and its derivative ejection fraction (EF) are crucial [9] in research as well as in clinical decision making. As cardiovascular magnetic resonance (CMR) has the highest reproducibility for RV volume measurement it is considered to be the gold standard [10] [11]. However, the assessment of EF is only one part of the functional evaluation and must be complemented with other parameters. Tissue characterization with CMR and the use of new functional measurements (myocardial deformation, strain) from either echocardiography or CMR help to understand mechanisms of ventricular adaptation and interaction. It is known from previous studies that in patients with congenital heart defect [1], both RV volume overload and RV pressure overload can lead to RV dilatation, decreased RVEF, and eventually RV failure [12] [13]. Causes of RV pressure overload that can lead to untimely RV failure include atrially repaired transposition of the great arteries (TGA) and congenitally corrected transposition (CCTGA). Understanding the mechanisms of the RV response to various loading conditions and mechanisms of interventricular interaction [14] [15] [16] is important in global assessment, but the exact mechanisms of adaptation and how adaptation affects different aspects of RV function remains largely unknown. Myocardial strain has been suggested to provide additional information about ventricular function, [17] however very few studies have investigated the specific adaptive mechanisms in conditions of CHD.

In the current study, we validated RV and LV strain and atrioventricular plane displacement (AVPD), as measured by CMR, against RV and LV volumes in patients with CHD.

Additionally, we investigated the correlations between strain and displacement with exercise capacity and oxygen uptake. As LV myocardial strain correlates with other measures of LV systolic function, we assumed that RV strain would correlate with measures of RV function in patients with tetralogy of Fallot (TOF) and TGA. [18]. These two conditions differ in terms of afterload. In the Fallot group, systolic RV pressure is normal or only slightly increased while in the TGA group the systolic RV pressure is on a systemic level. Thus, the relationship between EF and longitudinal and radial strain might differ between the two groups. We hypothesized that the RV response to increased afterload would be expressed as alterations in interventricular relationships affecting myocardial strain, chamber volume, and exercise capacity.

The study was carried out after approval from the Regional Ethical Review Board in Linköping (Registration number 2012/334-31) and in accordance with the Declaration of Helsinki. All patients provided written informed consent prior to their participation. The study was retrospectively registered as a clinical trial at: www.isrctn.com/ISRCTN18376089.

## Methods

### Patient population

**Fig 1:**
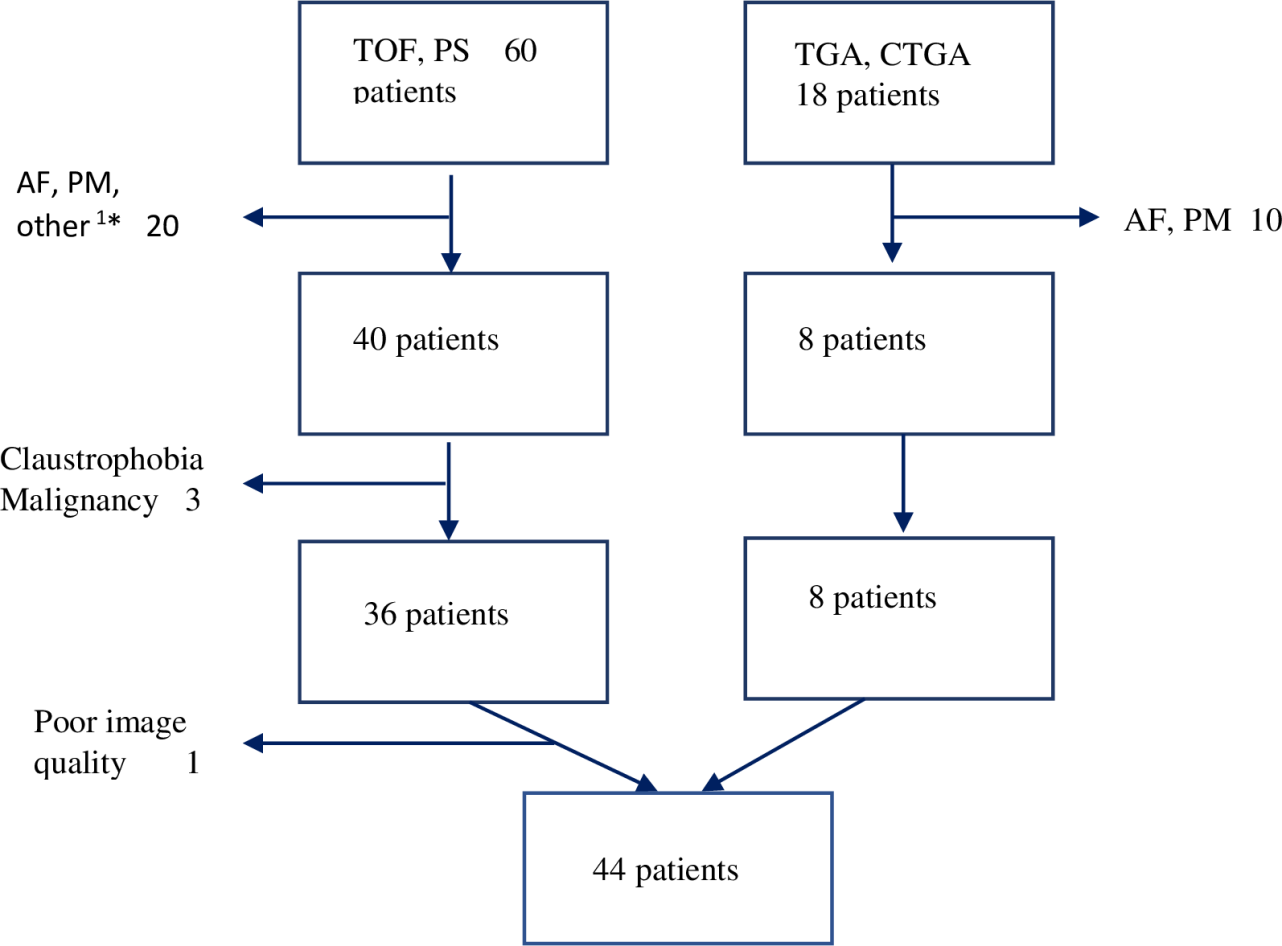
CONSORT flow chart for the inclusion and exclusion of study participant*s*. 1* inability to cooperate, AF atrial fibrillation, PM Pacemaker, TGA Transposition of the Great Arteries, CCTGA congenitally corrected transposition of the Great Arteries, TOF Tetralogy of Fallot, PS pulmonary stenos.

Fifty patients with CHD, followed as outpatients at the Linkoping University Hospital Cardiology Department were asked to participate in the study. Exclusion criteria were arrhythmia interfering with the CMR examination, the presence of a pacemaker, claustrophobia, and age less than seventeen years. As part of the routine examinations, all patients underwent echocardiography to exclude significant valvular regurgitation, except for regurgitation of the pulmonary valve. Forty-four patients (21 women) who met the eligibility criteria were enrolled from October 2013 to March 2015 while 6 declined to participate, Table 1 and Fig 1.

**Table 1:** Basic characteristics.

Variable	RV systemicposition N = 8	RV subpulmonic N = 36	pValue	ControlGroup N = 7
Age	36 (± 7)	34 (± 11)	ns	37(±14)
BMI kg/m^2^	26 (± 2.9)	24 (± 4)	ns	24 (± 3)
BSA m^2^	1.9 (± 0.26)	1.9 (± 0.25)	ns	1.9 (±0,2)
SBP mmHg	116 (± 14)	116 (± 14)	ns	120 (±10)
DBP mmHg	73 (± 8)	74 (± 12)	ns	75 (±9)
Systolic RV pressure	116 (± 14)	25 (± 10)	sign.	n/a
Heart rate beats/min	60 (± 6)	71 (± 13)	ns	65 (± 14)
QRS msec	105 (± 24)	139 (± 29)	0,004	98 (± 10)
NYHA FC I/II/III/IV (%)	38/50/12/0	61/23/16/0		100/0/0/0

BMI = Body mass index, BSA = Body surface area, SBP = systolic blood pressure, DBP = diastolic blood pressure, NYHA FC = New York Heart Association Function Class, (SD) = Standard deviation, RV = Right Ventricle, ns = non-significant, n/a = not available.

Thirty-six patients (47% female) were included in the group with the RV in the sub-pulmonic position, supporting the pulmonary circulation, with normal or only slightly elevated systolic pressure. This group included 29 patients with corrected TOF, five patients with recurrent pulmonary regurgitation (PR) after pulmonary valvulotomy, and two patients after corrected double-chamber RV. Nine patients presented with PR of moderate severity, while the remaining patients had either mild or no regurgitation at all. All patients had undergone surgical treatment. Twenty-three patients had surgery once, six patients twice, and seven patients three times. Surgical corrections were trans-ventricular in 20 patients, trans-annular in 12, and trans-atrial in two. Furthermore, patients in this group underwent two balloon dilatations, 14 pulmonary homograft replacements, one balloon and one open valvulotomy. In one case, details of the correction were not known. Mean age at the time for surgical correction in this group was 4 years.

The group with the RV in the systemic position (with systemic systolic blood pressure [BP]) consisted of five patients with TGA corrected ad modum Senning, one patient corrected according to Mustard, and two patients with CCTGA. In this group, four patients were male. Mean age at the time for initial surgery was 1.7 years.

The control group consisted of seven healthy volunteers that underwent CMR only. Five members of this group were male.

### CMR acquisition protocol and analysis

CMR was performed on a 1.5T scanner (Achieva Nova Dual, Philips Healthcare, Best, The Netherlands) equipped with a cardiac phased-array receiver coil. Cine images were obtained using a breath-hold segmented-k-space balanced fast-field echo sequence (SSFP) employing retrospective electrocardiogram (ECG) gating in the long axis two-chamber (2Ch) and four-chamber (4Ch) views and the apical long-axis view (3Ch) of the LV, as well as in a stack of short axis slices (slice thickness 8 mm, gap 2 mm) covering both ventricles from the base to the apex. Typical imaging parameters were: field of view = 320 mm, repetition time/echo time = 3.1 msec / 1.5 msec, flip angle 45^0^, and acquired matrix size 192x182 reconstructed to 256x256.

Analysis was performed on a workstation equipped with Segment v2.0 R5024, a semi-automatic software for cardiac analysis designed for i.a. volumetric analysis [19, 20].

**Fig 2:**
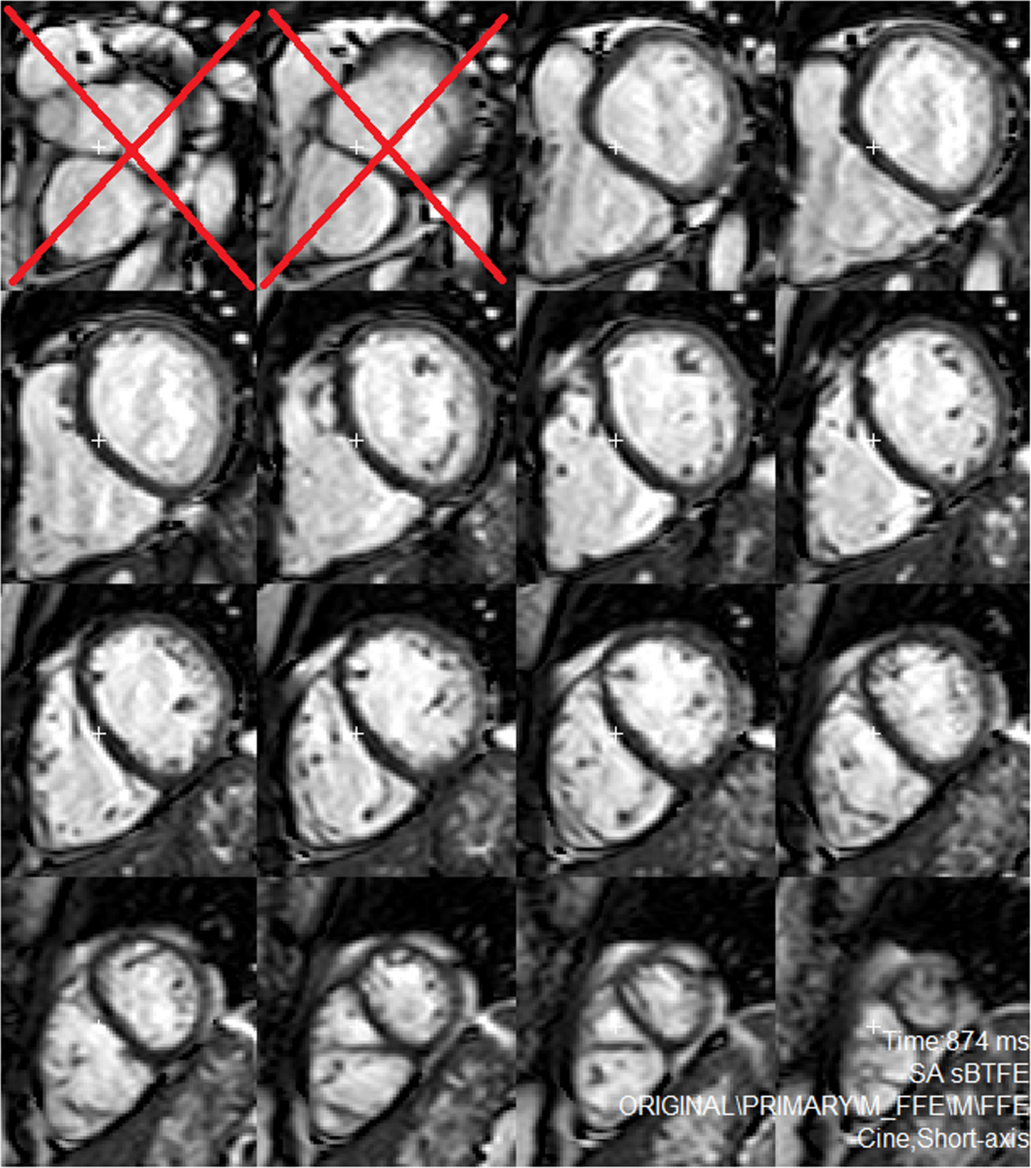
Example of segmentation of the shortaxis slices using segment. Slices that did not include >50% of the circumference of the myocardium were excluded from segmentation.

Volumes and EF for both ventricles were derived from short-axis slices after manually tracing the endocardial borders excluding the papillary muscles. End-diastole (ED) and end-systole (ES) were defined as the frames with the largest and the smallest ventricular slice area in relation to the ECG and the opening and closure of the aortic and AV valves. In case of discrepancies (e.g., long QRS duration in right or left bundle branch block morphology), the size of the ventricular area was the determining factor, i.e. maximum size was used to determine ED volume and minimum size was used to determine ES volume, Fig 2.

Global and regional longitudinal strain was calculated for both ventricles using a feature-tracking software (2D-CPA, Cardiac Performance Analysis v1.2, TomTec Imaging Systems, Germany). Global longitudinal strain (GLS) provided by the software was measured by calculating the average value of six segments in the LV and six segments in the RV obtained in the 4Ch view.

**Fig 3:**
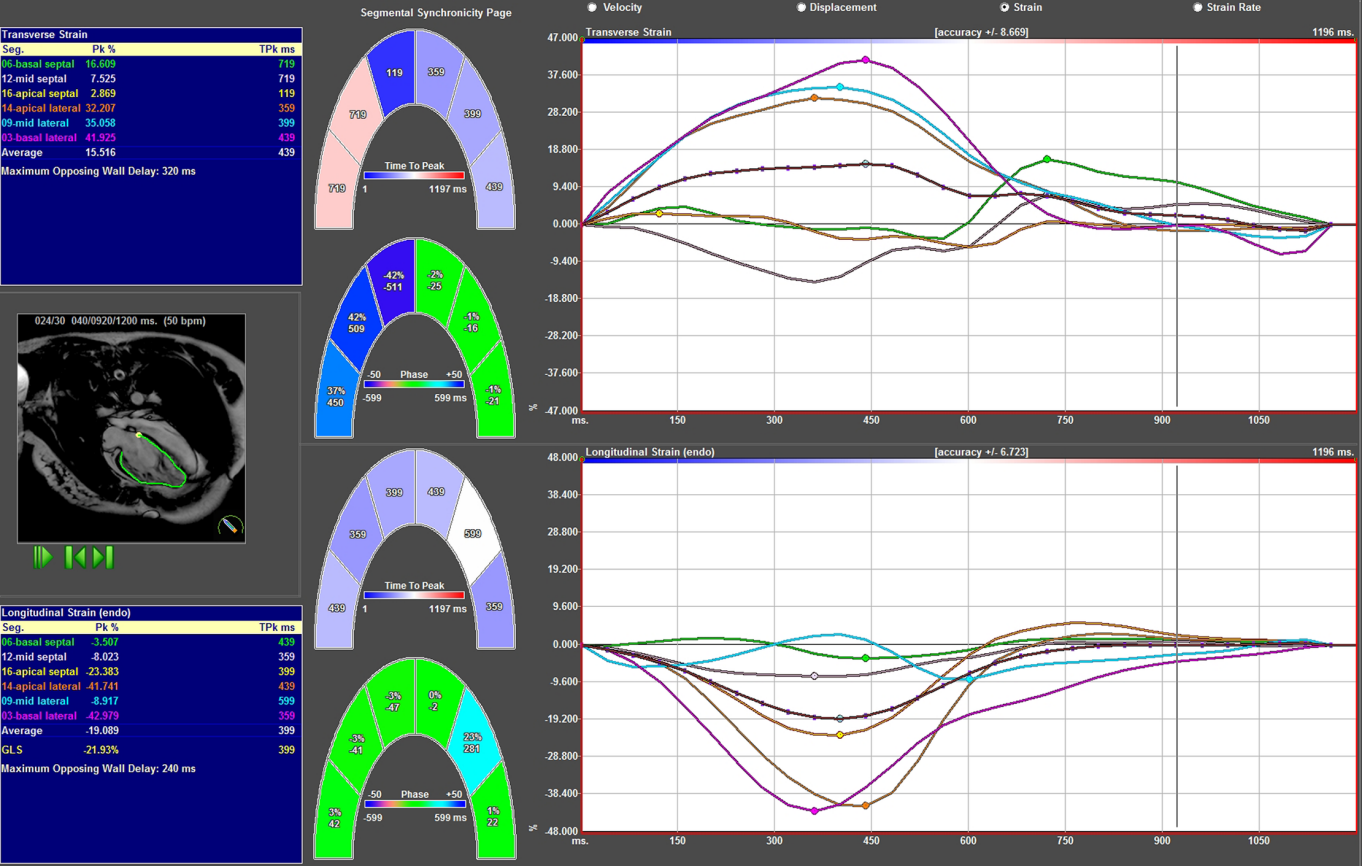
Results from strain analysis of TGA patient in the 4 Ch view. Segmental radial strain curves superior right, segmental longitudinal strain curves inferior right. Left superior blue box shows radial strain amplitude results and left inferior blue box longitudinal strain amplitude results. In the middle graphical display of results.

**Fig 4:**
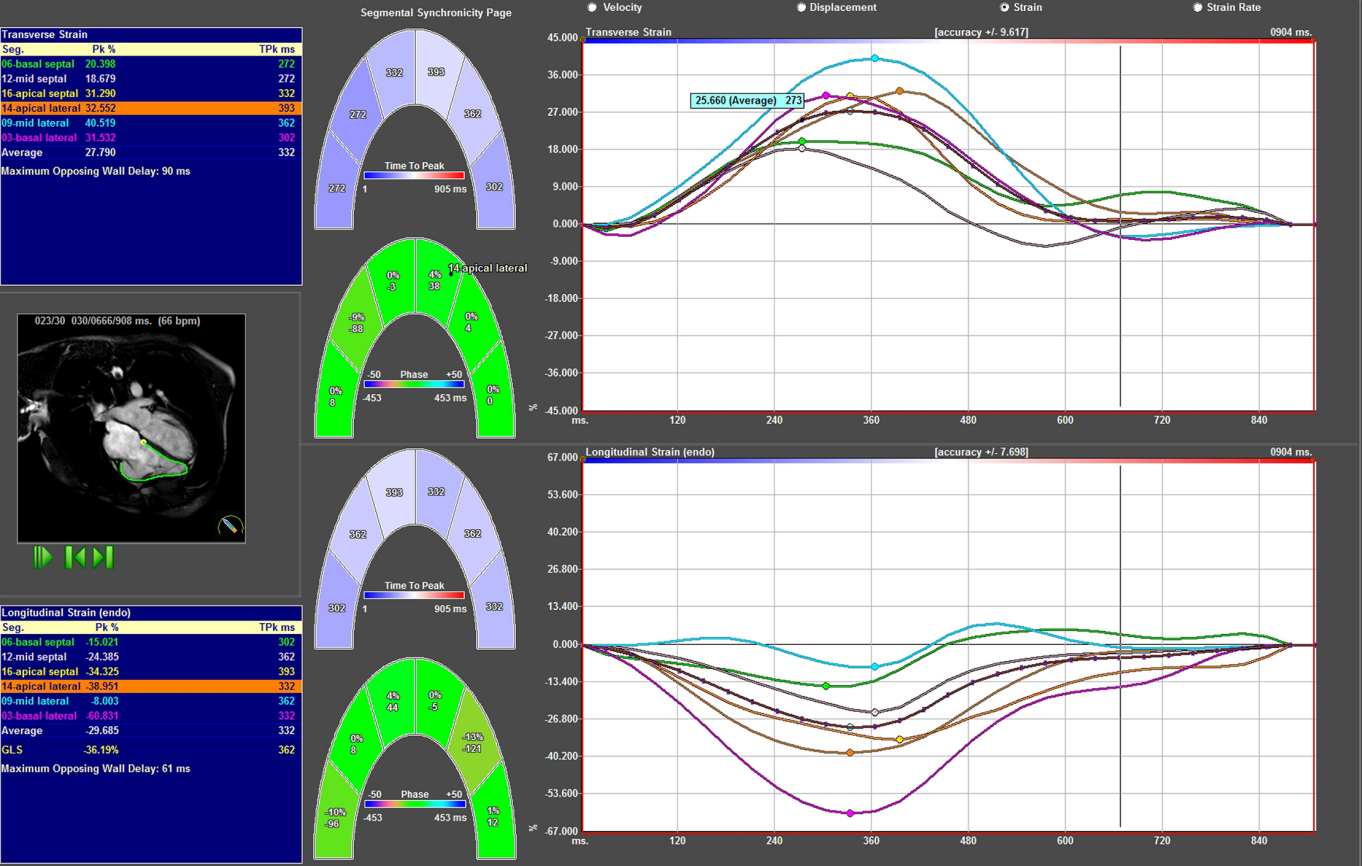
Results from strain analysis of fallot patient in the 4 Ch view. Segmental radial strain curves superior right, segmental longitudinal strain curves inferior right. Left superior blue box shows radial strain amplitude results and left inferior blue box longitudinal strain amplitude results. In the middle graphical display of results.

Tracking of the 4Ch view began with outlining the LV endocardium from the septal AV-plane in the clockwise direction and, for the RV, from the septal AV plane in the counter-clockwise direction, Fig 3 and Fig 4, S1 Video and S2 Video.

**Fig 5:**
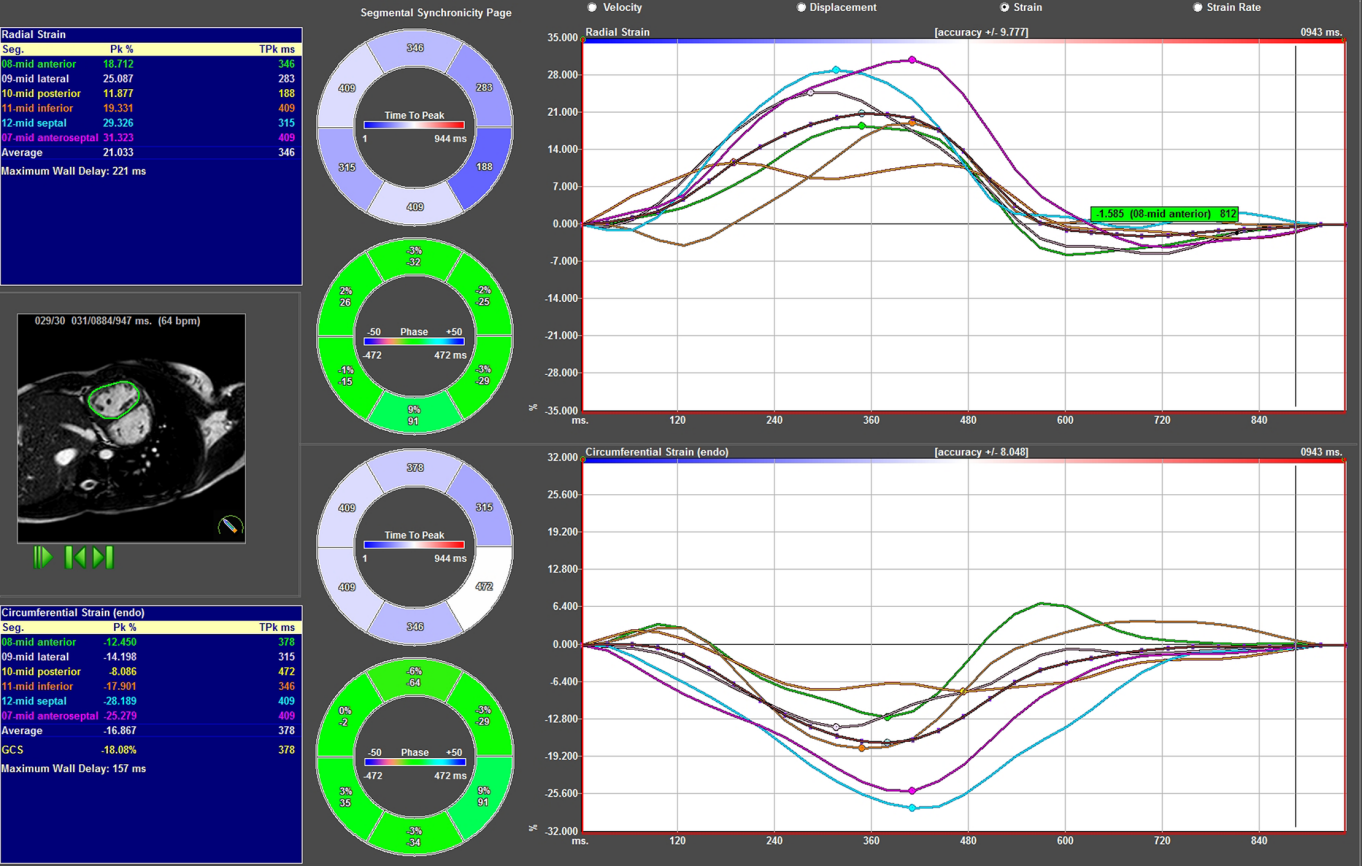
Results from strain analysis of TGA patient in the shortaxis view. Segmental radial strain curves superior right, segmental circumferential strain curves inferior right. Left superior blue box shows radial strain amplitude results and left inferior blue box circumferential strain amplitude results. In the middle graphical display of results.

**Fig 6:**
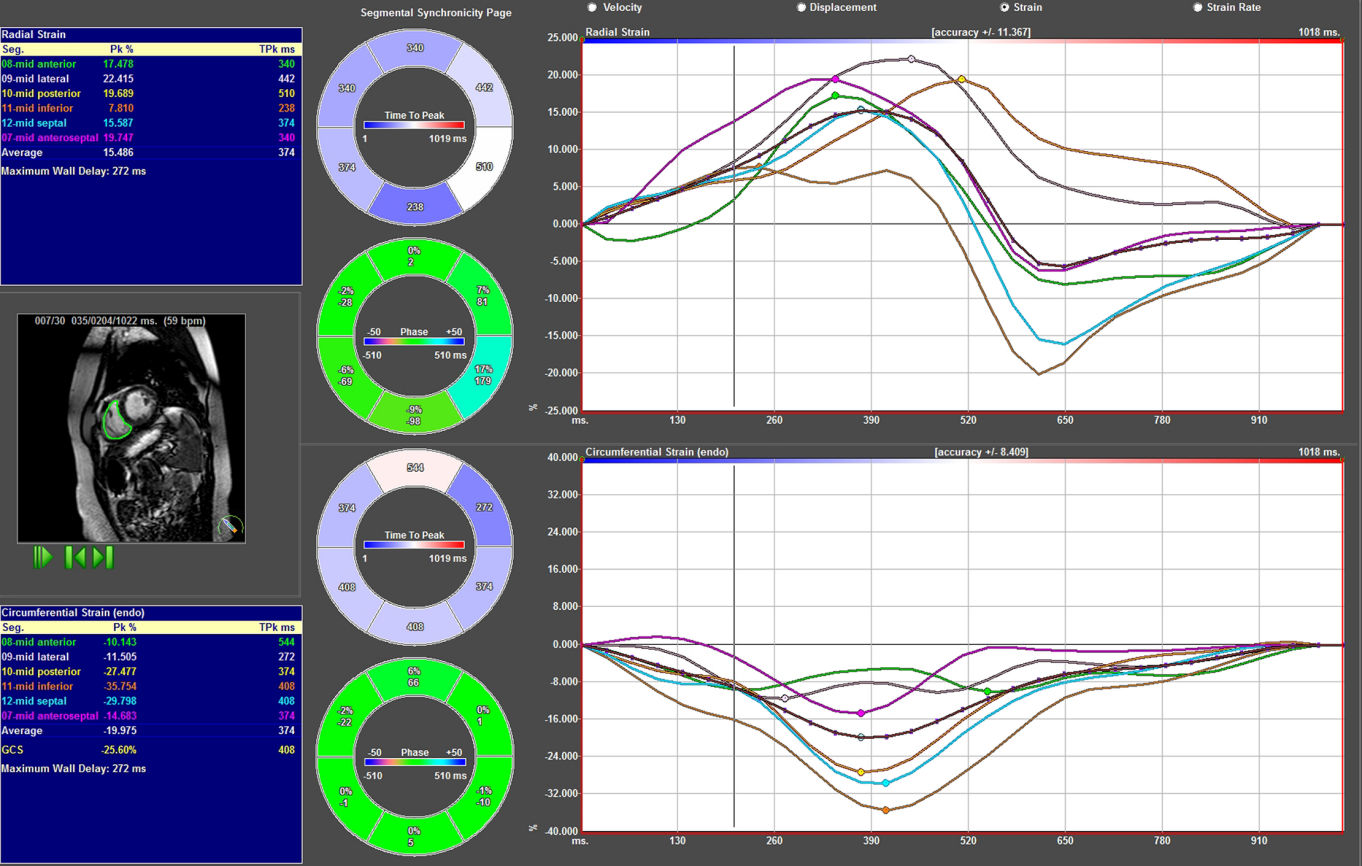
Results from strain analysis of fallot patient in the short axis view. Segmental radial strain curves superior right, segmental circumferential strain curves inferior right. Left superior blue box shows radial strain amplitude results and left inferior blue box circumferential strain amplitude results. In the middle graphical display of results.

Radial strain was analysed from tracking the middle slice of the short axis view at the midventricular level of the RV and LV, Fig 5 and Fig 6, S3 Video, S4 Video and S5 Video of normal reference.

In order to reduce variability, the mean of three repeated segmental measurements was used [21]. The analysis was performed offline on cine images that were previously anonymized. Radial displacement was not attempted since through-plane motion of the ventricles could not be accounted for.

### Cardiopulmonary exercise test

In all study patients, a maximum symptom-limited (perceived exertion ≥ 17/20 according to the “Borg” rating scale [22], cardiopulmonary exercise test (CPET) was performed on a cycle ergometer (Monark Ergomedic 839E, Monark Exercise AB, Vansbro, Sweden) with the patient in an upright position. Depending on the expected individual physical work capacity, a protocol was devised that began with 5 minutes of steady-state load at 30/50 W followed by load increases in increments of 10/20 W/min with the goal of reaching maximal exercise capacity within 8–12 minutes. Breath-by-breath respiratory gas analysis was performed using a Jaeger Oxycon Pro (Vyaire Inc., Mettawa, IL, USA). Peak VO_2_ was calculated from the mean of values measured during the last 60 s of exercise and was expressed as ml x kg^-1^ and ml x kg^-1^ x min ^-1^. Patients were considered to have achieved maximal exercise capacity if the respiratory exchange ratio exceeded 1 continuously for three minutes or longer. Patients were evaluated by continuous 12-lead ECG monitoring, and BP was measured using a cuff manometer at rest and at three minute intervals during exercise. Spirometry was performed in all patients immediately before the CPET to obtain forced vital capacity and forced expiratory volume in one second measurements.

### Statistical analysis

All continuous data and values are presented as the means ± standard deviation (SD) and as median (min-max). Student’s t-test was used for comparison between the two study groups (TGA patients and TOF patients) and between a study group and the control group. A test of equal variance for comparison between groups and further calculations were performed and the degrees of freedom were calculated according to Satterthwaite in case of significant differences in variance or by pooled standard error in the case of presumptive equal variance.

For strain regression analysis, a multiple linear regression model was fitted for those measurements that in univariate analysis differed significantly between groups. The regression model contained four parameters of exercise capacity as explanatory variables and was fitted to radial LV and RV strain, RVGLS, LVEF, and RVAVPD as response variables. The correlation between measurements was evaluated using the Pearson correlation coefficient. Strain results were depicted in graphs with box plots and linear regression to represent the correlation between AVPD and the measurements used to assess exercise capacity.

SAS software v9.4 and SPSS Statistics v23 were used for the statistical analyses.

## Results

### Patient population

Age, body mass index (BMI), body surface area (BSA), BP, workload, and peak oxygen uptake did not differ between groups. RV end-diastolic volume (RVEDV) in the TGA group was 220±82 ml (mean ± SD) and 197±56 ml in the TOF group. The difference in volume between the two groups was not significant. The mean RV systemic systolic BP in TGA patients was 116mmHg (± 14). In the group with the sub-pulmonic RV, mean systolic RV pressure was 25 mmHg (± 10), based on the maximal velocity of the systolic regurgitant flow across the tricuspid valve, except for seven TOF patients, in whom systolic RV pressure was calculated based on the maximal systolic velocity across the pulmonary valve. In the resting ECG, the QRS duration was longer in the TOF group than in the group with the pressure-loaded RV, Table 2.

**Table 2:** Descriptive data.

Variable	RV TGA N = 8 (SD)	RV TOF N = 36 (SD)	P value comparison TGA and TOF	Control N = 7
LV EDV ml			ns	
Mean (SD)	153 (± 57)	156 (± 31)		190 (± 35)
Median (min-max)	145 (86–235)	155 (107–222)		196 (141–229
LV ESV ml			ns	
Mean (SD)	71 (± 34)	80 (± 18)		92 (± 26)
Median (min-max)	60 (35–129)	78 (51–122)		82 (67–138)
LV EF %			0.009	
Mean (SD)	55 (± 7)	49 (± 6)		54 (± 6)
Median (min-max)	57 (40–62)	50 (37–60)		53 (48–61)
RV EDV ml			ns	
Mean (SD)	220 (± 82)	197 (± 56)		
Median (min-max)	167 (146–350)	182 (111–339)		
RV EDVi ml			ns	
Mean (SD)	113 (± 32)	106 (±24)		112 (±26)
Median (min-max)	103 (80–173)	102 (65–164)		108 (80–148)
RV ESV ml			ns	
Mean (SD)	129 (± 61)	114 (± 41)		101 (± 25)
Median (min-max)	98 (69–246)	107 (62–260)		108 (63–128)
RV EF %			ns	
Mean (SD)	43 (± 6)	43 (± 8)		49 (± 4)
Median (min-max)	43 (30–53)	43 (23–60)		48 (44–55)
RVAVPD mm			0.000	
Mean (SD)	8 (± 1)	11 (± 3)		16 (± 2)
Median (min-max)	8 (7–10)	11 (6–17)		15 (15–19)
LVAVPD mm			ns	
Mean (SD)	10 (± 3)	11 (± 2)		15 (± 4)
Median (min-max)	9 (6–15)	11 (8–14)		16 (11–20)
Peak O_2_ uptake ml/(kgxmin)			ns	n/a
Mean (SD)	23 (± 5)	27 (± 7)		
Median (min-max)	23 (16–31)	27 (15–42)		
Max work load Watt			ns	n/a
Mean (SD)	160 (± 51)	167 (± 61)		
Median (min-max)	164 (96–226)	155 (69–294)		
Global long RV strain %			ns	
Mean (SD)	-17 (± 4)	-20 (± 6)		-20 (± 4)
Median (min-max)	-16 (10–20)	-19 (11–36)		-20 (15–26)
Global long LV strain %			ns	
Mean (SD)	-23 (± 7)	-21 (± 5)		-20 (± 4)
Median (min-max)	-24 (13–31)	-20 (14–34)		-20 (15–26)

CMR = Cardiac Magnetic Resonance, TGA = Transposition of the Great Arteries, LV = left ventricle, RV = right ventricle, EDV = end diastolic volume, EDVi = indexed end diastolic volume; ESV = end systolic volume, EF = ejection fraction, AVPD = atrioventricular plane displacement, Peak O_2_ uptake = peak Oxygen uptake, Global long RV strain = global longitudinal right ventricular strain, Global long LV strain = global longitudinal left ventricular strain, Radial mid RV strain radial = midventricular right ventricular strain, Radial mid LV strain = Radial midventricular left ventricular strain, ns = nonsignificant, n/a = not available, SD = standard deviation

### Reproducibility

Reproducibility of right ventricular volume measurements was calculated from segmentations performed by two independent operators on sixteen randomly selected patients. The result shows very god reproducibility concerning all measured parameters (end diastolic volume, end systolic volume and ejection fraction) with interobserver ICC for average measurement of RVEDV 0.96, for RVESV 0.97 and for EF 0.78 (lower due to being calculated as a ratio with twice as many underlying measurements).

### Transposition of the great arteries

In this group RVGLS (-17% ± 4), RVEF (43% ± 6), and the displacement of both left (10 mm ± 3) and right (8 mm ± 1) atrioventricular planes were significantly lower than in controls (RVGLS -28% ± 7), (RVEF 49% ± 4) and (LVAVPD 15 mm ± 4, RVAVPD 16 mm ± 2), respectively, Table 3

**Table 3:** Comparison between the TGA group and the controls.

Order	Variable	MeanDifference TGA–control	Lower CL Mean	UpperCL Mean	t-value	Pr > |t|
1	Radial mid RVstrain %	3.07	-3.67	9.82	0.98	0.343
2	Radial mid LVstrain %	-21.73	-31.05	-12.42	-5.08	0.000
3	Global long RVstrain %	-11.64	-16.91	-6.37	-4.74	0.000
4	Global long LVstrain %	2.45	-3.69	8.60	0.86	0.404
5	RV EF %	-6.11	-12.03	-0.19	-2.23	0.044
6	LV EF %	0.98	-6.22	8.19	0.29	0.773
7	RV EDV ml	25.48	-48.83	99.79	0.74	0.472
8	RV ESV ml	28.41	-24.89	81.71	1.21	0.255
9	LV EDV ml	-36.82	-90.75	17.11	-1.48	0.164
10	LV ESV ml	-22.23	-56.80	12.34	-1.39	0.188
11	RV AVPD mm	-8.05	-9.59	-6.50	-11.27	0.000
12	LV AVPD mm	-5.39	-8.80	-1.98	-3.41	0.005

CMR = Cardiac Magnetic Resonance, TGA Transposition of the Great Arteries, Radial mid RV strain radial = midventricular right ventricular strain, Radial mid LV strain = Radial midventricular left ventricular strain, Global long LV strain = global longitudinal left ventricular strain, Global long RV strain = global longitudinal right ventricular strain, RV = right ventricle, LV = left ventricle, EDV = end diastolic volume, ESV = end systolic volume, AVPD = atrioventricular plane displacement

In the group with the RV in systemic position, the only positive correlation was between RVEF and radial RV strain (r = 0.89 p<0.01). No other interventricular correlations between various strain aspects and volumetric measurements were found.

### Tetralogy of fallot

In this group, radial LV strain, as well as LVEF (49% ± 6) and biventricular AVPD were reduced in comparison with those in the control group (p<0.05, p<0.05, and p<0.000, respectively), Table 4.

**Table 4:** Comparison between TOF group and control.

Order	Variable	Mean difference TOF–control	Lower CL Mean	Upper CL Mean	t-value	Pr > |t|
1	Radial mid RVstrain %	-0.64	-4.89	3.61	-0.30	0.764
2	Radial mid LVstrain %	-7.94	-15.13	-0.76	-2.23	0.031
3	Global long RVstrain %	-8.09	-12.57	-3.61	-3.64	0.001
4	Global long LVstrain %	0.76	-3.15	4.68	0.39	0.695
5	RV_EF %	-5.77	-11.74	0.19	-1.95	0.058
6	LV_EF %	-5.48	-10.26	-0.69	-2.31	0.026
7	RV_EDV ml	2.33	-42.60	47.26	0.10	0.917
8	RV_ESV ml	13.59	-19.26	46.44	0.84	0.408
9	LV_EDV ml	-33.79	-60.02	-7.57	-2.60	0.013
10	LV_ESV ml	-13.27	-29.28	2.73	-1.67	0.102
11	RV_AVPD mm	-5.68	-7.70	-3.66	-5.67	0.000
12	LV_AVPD mm	-4.64	-6.29	-2.99	-3.47	0.012

CMR = Cardiac Magnetic Resonance, TOF = Tetralogy of Fallot, Radial mid RV strain radial = midventricular right ventricular strain, Radial mid LV strain = Radial midventricular left ventricular strain, Global long LV strain = global longitudinal left ventricular strain, Global long RV strain = global longitudinal right ventricular strain, RV = right ventricle, LV = left ventricle, EDV = end diastolic volume, ESV = end systolic volume, AVPD = atrioventricular plane displacement.

In the TOF group, we found a significant correlation between LVEF and RVEF (r = 0.58, p<0.01), between LVGLS and RVGLS (r = 0.58, p < 0.01), and between LVAVPD and RVAVPD (r = 0.64, p<0.01).

### Comparison between groups

**Fig 7:**
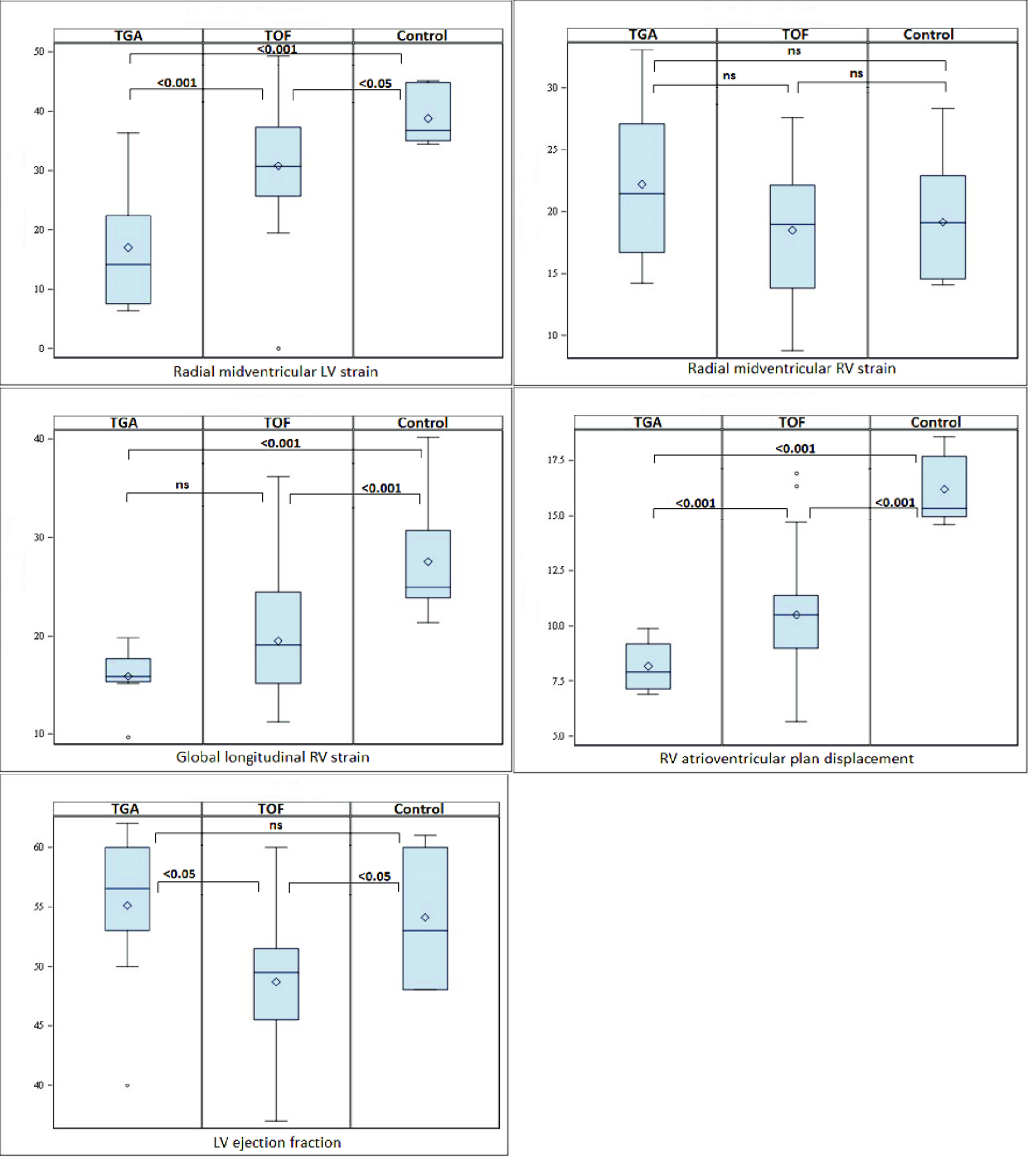
Boxplots for LVEF, radial LV strain, global longitudinal RV strain, RV AVP displacement for three different groups. TGA group, Fallot group and Control group. TGA = Transposition of the Great Arteries, LV = left ventricle, RV = right ventricle, AVP atrioventricular plane, * = significant difference, ns = non-significant difference.

RV wall thickness was significantly increased in the group with the systemic right ventricle comparing to the Fallot group, 5.3mm (±0.8 mm) versus 3.5 mm (± 0.5 mm) p < 0.000. In a comparison between the two study groups, we found significant differences in LV radial strain, LVEF, and RVAVPD. RVAVPD was significantly lower in patients with a systemic RV compared to TOF patients (p<0.000) and for both groups compared with the control group. Radial LV strain was significantly lower in the group with systemic RV pressure compared to that in the TOF group (p<0.01) and in the control group (p<0.000). The cineloops demonstrate different contraction patterns in the three groups. In patients with systemic RV pressure, radial RV strain tended to be higher while RVGLS tended to be lower (less negative) than in patients with TOF, but the difference was not significant. In comparison with the control group, the impairment in RVGLS was significant for both study groups (p<0.001). This finding was consistent with the significantly depressed value of the RVAVPD in the TGA population (p<0.000), Fig 7.

**Fig 8:**
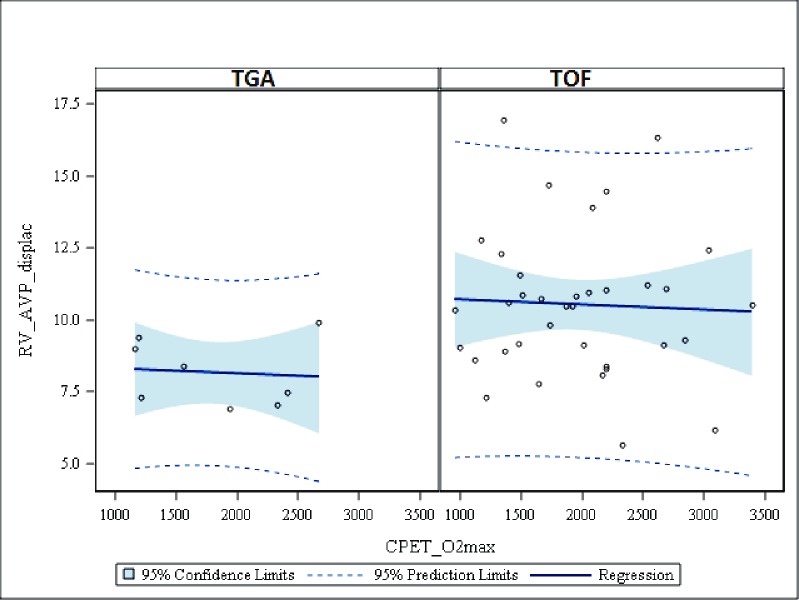
Scatter plots with regression lines for RV AV plane displacement versus CPET-variables. **Maximal workload in Watt.** CPET = Cardiopulmonary exercise test, W max = max workload in Watt, RV_AVP_displac = Right ventricular atrioventricular plane displacement, TGA = Transposition of the Great Arteries.

**Fig 9:**
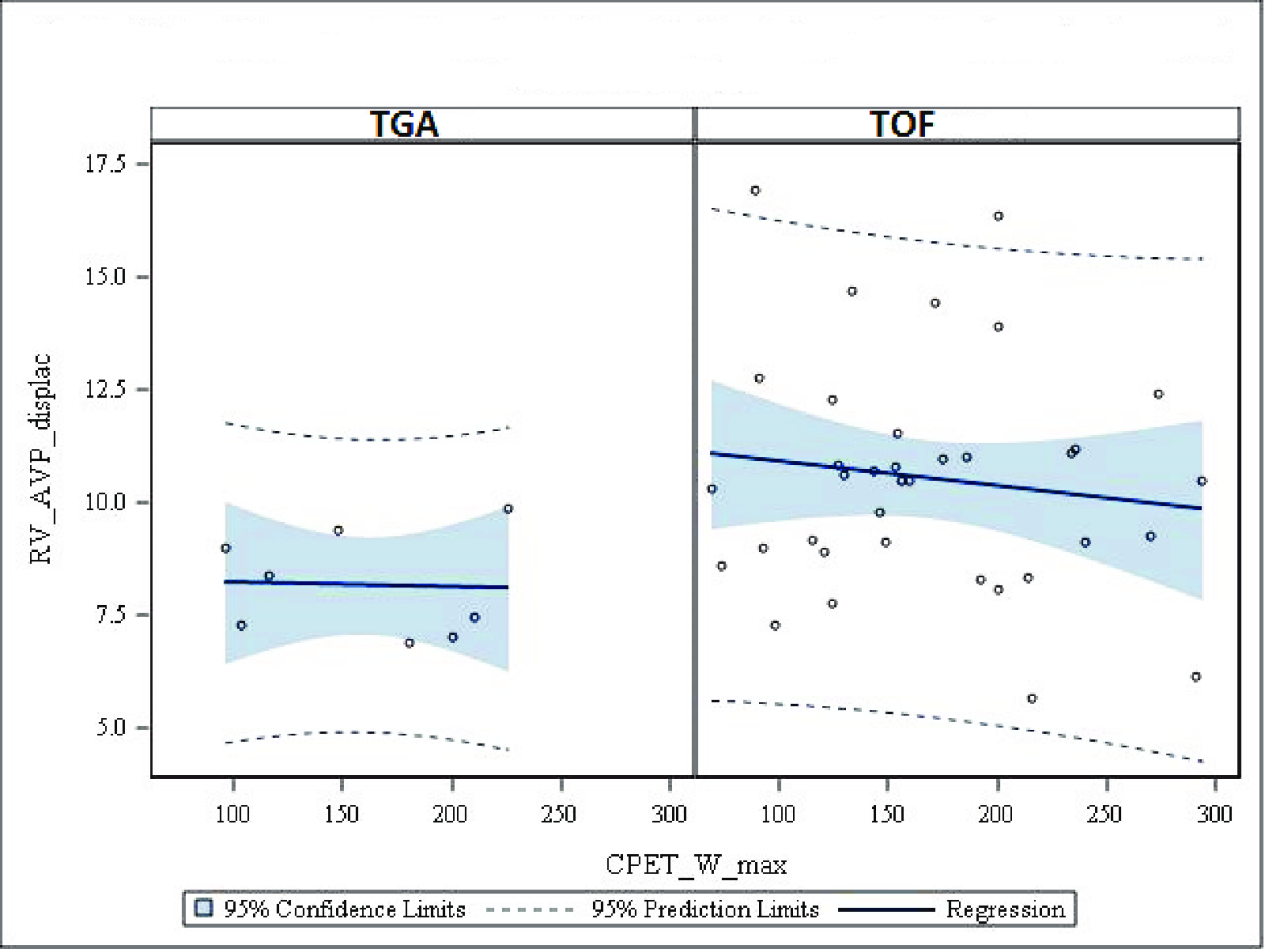
Scatter plots with regression lines for RV AV plane displacement versus CPET-variables. **Maximal Oxygen uptake.** CPET = Cardiopulmonary exercise test, O2max = maximal Oxygen uptake, RV_AVP_displac = Right ventricular atrioventricular plane displacement, TGA = Transposition of the Great Arteries.

Regression analysis showed that RVAVPD could not predict exercise capacity expressed in W or oxygen uptake in either TGA or TOF patients, Figs 8 and 9.

## Discussion

Various aspects of RV and LV strain differ between the disease groups. We observed differences of radial and longitudinal strain [23] when comparing each study group with the control group as well as when comparing the study groups to each other.

RVAVPD and LVAVPD (measured with CMR) were significantly reduced in both study groups in comparison with the healthy control group. [24]

We interpret this finding to be the result of the underlying cardiac disease as well as an effect of surgery performed to correct abnormalities [25].

A reduction in AVPD requires a compensatory increase in radial shortening to maintain stroke volume. In a comparison between the two groups of patients, RVAVPD was significantly lower in patients with the RV in the systemic position. This is consistent with findings in LV hypertrophy where displacement of the mitral ring (MAPSE) is reduced [26] and the correlation between LVEF and MAPSE is lost [27]. A reduction in RVAVPD could be reflected in longitudinal strain reduction, as found here.

### Relationship between radial strain and afterload

In the LV, radial strain was significantly lower in both study groups of patients as compared to the control group, with the lowest values found in those patients with the LV in the sub-pulmonic position (TGA group), the result of adaptation to diminished afterload possibly via a change in myocardial architecture [23].

In the case of the morphologic RV in the systemic position, the situation was the opposite. Radial RV strain was higher in that group of patients than in the TOF group, possibly a result of increased wall thickness in response to a high-pressure load, even if the relationship between strain and wall thickness failed to reach significance (p<0.07) possibly due to the small sample size. The changes observed may express ventricular adaptation to an altered afterload. The systemic RV mimics the morphology of the systemic LV by increasing radial mid-wall contraction. The correlation between radial strain and EF suggests a change in the pattern of contraction with a reduced role of the longitudinal component in preserving cardiac output.

### Effects of volume on contraction patterns

In the current study, RVEDV was similar in the two patient groups. The dilatation of the sub-pulmonic RV in TOF patients has been suggested to be the effect of multiple operations with patches as well as prolonged periods of pressure overload before surgical repair. Dilatation of the RV in the systemic position appears to be a progressive remodelling process aimed at preserving RV stroke volume, attributable to the increase in cardiac myocyte length [28]. Hypertrophy and dilatation causes the RV shape to become more spherical, with an enlarged cross-sectional area and flattening of the interventricular septum [28]. The absence of interventricular correlations between strain and EF in the sub-aortic and sub-pulmonic ventricles in the TGA group suggests that the role of the sub-pulmonic ventricle in the preservation of cardiac output in this group is reduced.

RVGLS was significantly decreased in both study groups in comparison with the control group. This is consistent with findings in the LV, where larger ventricles due to geometric assumptions have lower strain values [29]. RVGLS tends to be lower in the group with high systolic pressure and in patients with a hypertrophied systemic RV, which is also consistent with LV findings [30]. Moreover, the observed biventricular correlations between GLS, EF, and AVPD are consistent with previous observations on interventricular dependency in the TOF population [31].

### The interaction between cardiopulmonary exercise capacity and RVAVPD

The reduction in peak oxygen uptake demonstrated in the current study was comparable to previous findings by Kempny *et al*. [32]. Regression analysis for RVAVPD and CPET measurements revealed that peak oxygen uptake was not related to RVAVPD in either study groups, in line with previous findings [33].

In other words, increase in pumping by an increase in cardiac output and oxygen uptake must depend on changes in RV mechanics other than longitudinal shortening, e.g., changes in RV radial shortening or in interventricular interactions.

Transverse, or radial, strain has been shown to predict exercise capacity in TGA patients [34]. However, we were not able to confirm this finding, possibly due to our study’s low sample size.

### Limitations

The extensive and variable surgical treatment that study participants underwent complicated the interpretation of differences in cardiac pumping. Strain analysis depends on the accurate tracking of features in the cine images and the tracking is at times suboptimal which allows global assessment but prevents segmental analysis. The delineation of the right ventricular myocardium in patients with corrected congenital anomalies discussed in the current study is difficult. This limits the feasibility of the methods (both strain and volume measurements) and challenges the reliability of the results. However, in experienced hands, strain measurements have high consistency and reproducibility, as proven in our study with ICC above 0.9 for both inter and intra-observer measurements. Despite potential problems selecting the end-diastolic frame when the ECG displays a bundle branch morphology which is common with congenital abnormalities, the reproducibility of the volume measurements was also very good. Larger patient populations and longitudinal follow up are needed to determine the predictive value of strain and AVPD in the population of patients with congenital heart disease.

Although the group with systemic RV pressure overload was relatively small it was homogeneous and all studied patients were in stable clinical condition, most of them in NYHA functional class I or II. The control group was limited to only seven individuals but we obtained similar strain results as was found in the normal population of Takeuchi (RV strain) [35] and Maceira (LV strain) [36].

## Conclusions

This study demonstrates that differences in RV and LV longitudinal strain and AVPD, correlate with the degree of pressure load. This observation can be related, in part, to ventricular adaptation to altered loading conditions. These findings are at least partly consistent with those of previous studies describing adaptation processes in terms of increases in circumferential and decreases in the longitudinal deformation in the systemic RV [37].

The current study suggest that radial RV strain could be higher in the pressure loaded RV however the difference was not significant and further studies with larger patient population are needed to assess such an adaptation mechanism.

### Clinical applications

The present study shows that EF does not correlate with functional capacity in the studied patient population. While adverse remodelling takes place, EF is frequently preserved for a long time to maintain cardiac output. The focus in further research should be on measurements that indicate when adaptive remodelling progresses to cardiac failure. This knowledge is necessary for decisions on therapy as well as to aid in identifying the optimal time for heart transplantation.

## Supporting information

S1 Video (MP4)

S2 Video (MP4)

S3 Video (MP4)

S4 Video (MP4)

S5 Video (MP4)

## Abbreviations

2CH view: two chamber view
3CH view: three chamber view
4CH view: four chamber view
AVPD: trioventricular plane displacement
BP: Blood Pressure
BSA: Body surface area
BMI: Body mass index
CCTGA: Congenitally corrected transposition of the great arteries.
CHD: Congenital heart defects
CMR: Cardiac Magnetic Resonance
CPET: Cardiopulmonary exercise test
ED: End diastole
ES: End systole
EDS: End-systolic volume
EDV: End-diastolic volume
EDVi: End-diastolic volume indexed
EF: Ejection fraction
GLS: Global longitudinal strain
LV: Left ventricle
MAPSE: Mitral annular plane systolic excursion
NYHA: New York Heart Association
PR: Pulmonary regurgitation
RV: Right ventricle
SD: Standard deviation
SSFP: Steady-state free precession
TGA: Transposition of the Great arteries
TOF: Tetralogy of Fallot
